# Neck Sinus Post-Thyroidectomy Secondary to Retained Oxidized Regenerated Cellulose: A Case Report

**DOI:** 10.7759/cureus.37605

**Published:** 2023-04-15

**Authors:** Houssein Haidar Ahmad, Rana Ibrahim, Abbas Fadel

**Affiliations:** 1 Surgery Department, Saint George Hospital, Beirut, LBN; 2 Research Department, Saint George Hospital, Beirut, LBN; 3 Infectious Diseases Department, Saint George Hospital, Beirut, LBN

**Keywords:** paratracheal space, oxidized regenerated cellulose, deep neck fluid collection, chronic neck sinus, thyroidectomy

## Abstract

The present study aims to report the first case of chronic neck sinus post-thyroidectomy caused by oxidized regenerated cellulose (ORC). A 55-year-old female patient underwent a total thyroidectomy operation. Three months after the surgery, the patient presented with persistent purulent discharge and sinus at the site of the drain. A CT scan of the neck showed a fistula tract, deep-neck fluid collection, and bilateral paratracheal high-density lesions at the thyroid bed, suggesting infected foreign bodies. The patient underwent surgery, during which the mesh of the ORC was found nonresorbed at the paratracheal space. The treatment involved neck exploration with the removal of all retained material and excision of the sinus tract. The patient had a favorable outcome following the surgical excision of the sinus tract and the removal of retained hemostatic materials. Further research is needed to explore the risk factors and preventive measures for neck sinus formation to enhance the safety and outcomes of thyroidectomy.

## Introduction

Thyroidectomy is a common surgical procedure, and it is associated with potential complications such as bleeding, infection, hypocalcemia, and recurrent laryngeal nerve injury [1]. Chronic neck sinus formation following thyroidectomy is a rare but well-recognized complication, with few reported cases in the literature [2,3]. Retained foreign bodies, including nonabsorbable or nonresorbable sutures, and infections are the most common etiologies of this complication [2,4]. We report a case of chronic neck sinus formation caused by oxidized regenerated cellulose (ORC) after thyroidectomy, which, to the best of our knowledge, has not been previously reported. ORC is a commonly used hemostatic agent during various surgical procedures, including thyroidectomy, with an expected resorption time of two weeks [5]. The management of this complication typically involves exploration of the neck, removal of retained infected material, and excision of the sinus [2,6]. Identifying the underlying etiology is crucial to prevent further complications and ensure optimal patient care.

## Case presentation

**Figure 1:**
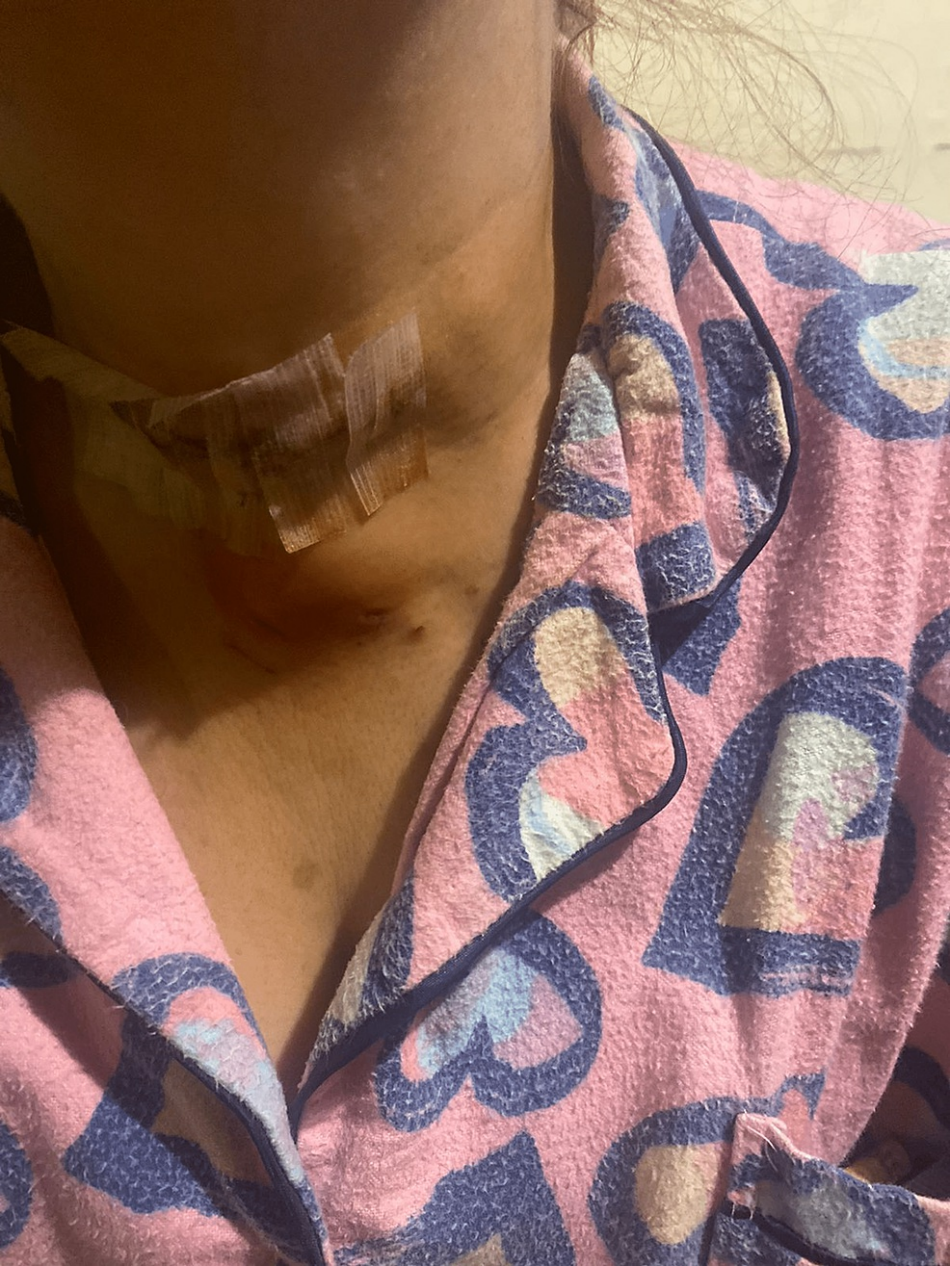
Swelling at the site of drainage

**Figure 2:**
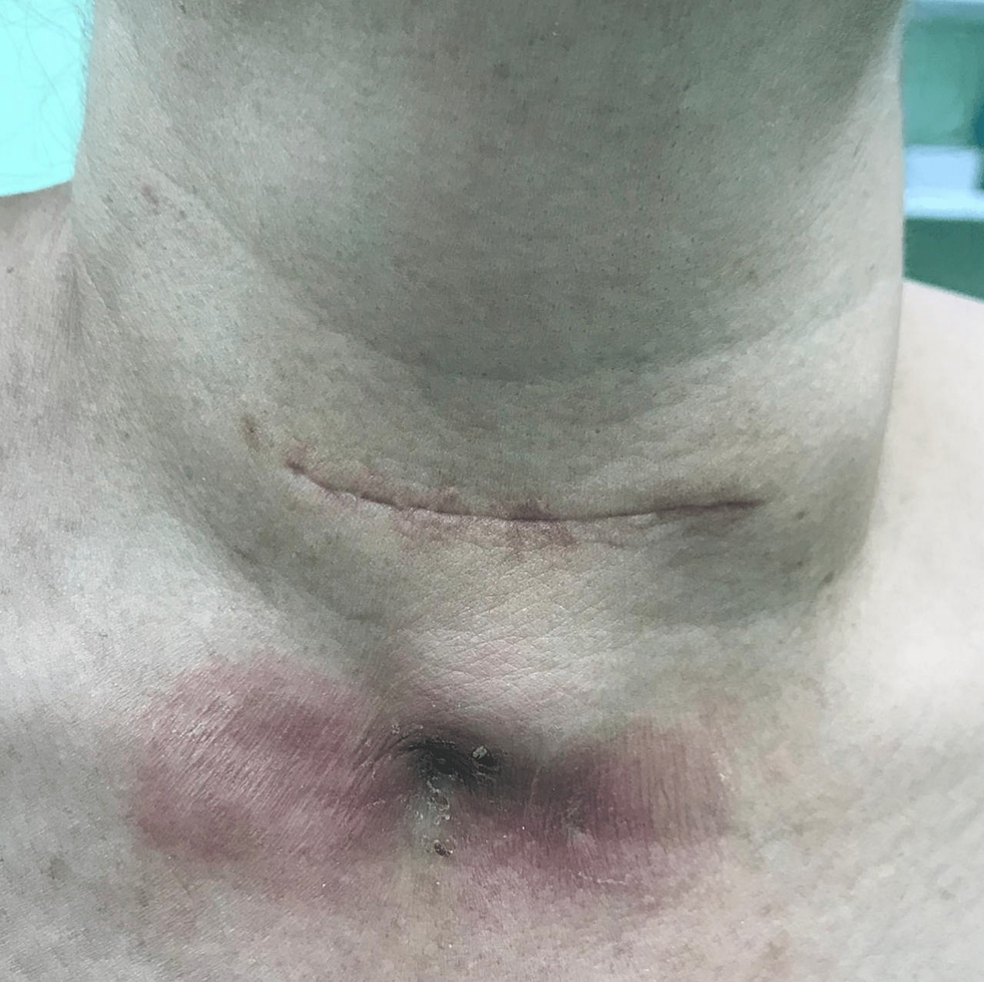
Purulent discharge at the site of drainage

**Figure 3:**
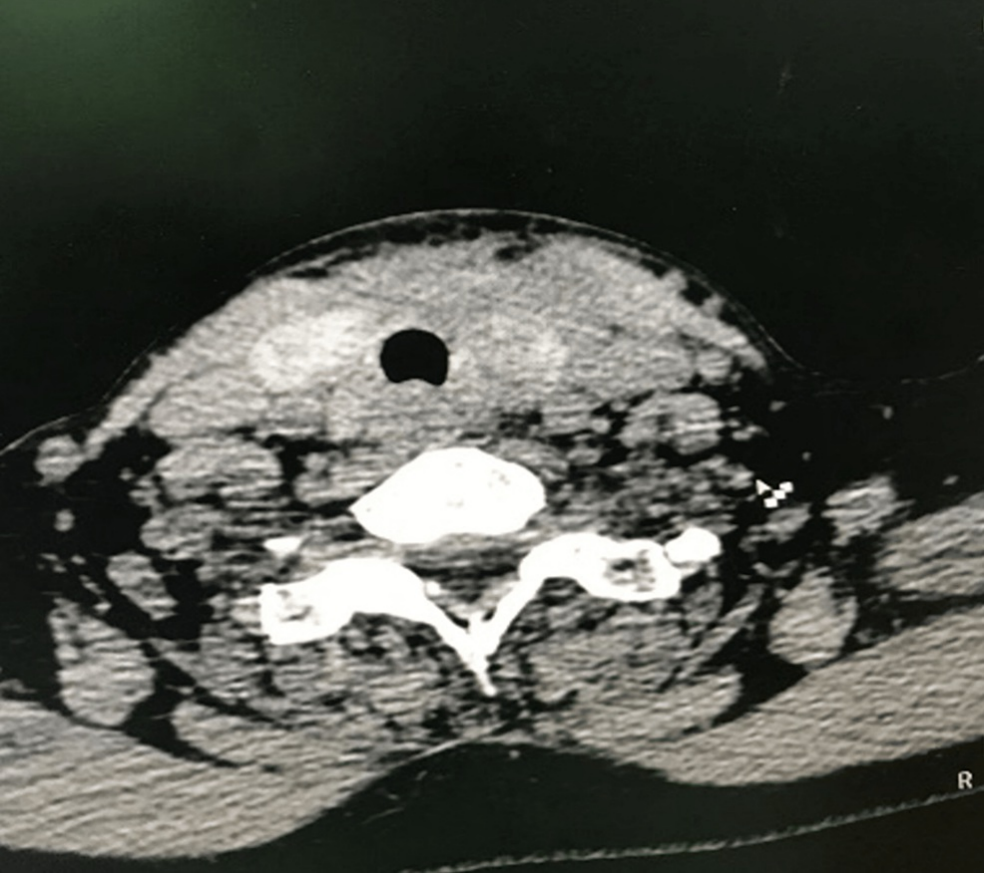
CT scan of the neck The image showed high-density lesions at the thyroid bed CT: computed tomography

A 55-year-old female patient was admitted to Saint George Hospital and diagnosed with toxic multinodular goiter and Graves' disease. The patient underwent total thyroidectomy surgery. After the surgery, a collection was noted at the drain site one week postoperatively (Figure 1). The collection was percutaneously drained under ultrasound guidance, and the fluid culture showed no growth of any microorganisms. Despite a prolonged course of antibiotics, the patient presented three months post-surgery with persistent purulent discharge and a sinus at the drain site (Figure 2). A CT scan of the neck (Figure 3) revealed the presence of a fistula tract, deep-neck fluid collection, and bilateral paratracheal high-density lesions at the thyroid bed, which were suggestive of infected foreign bodies.

**Figure 4:**
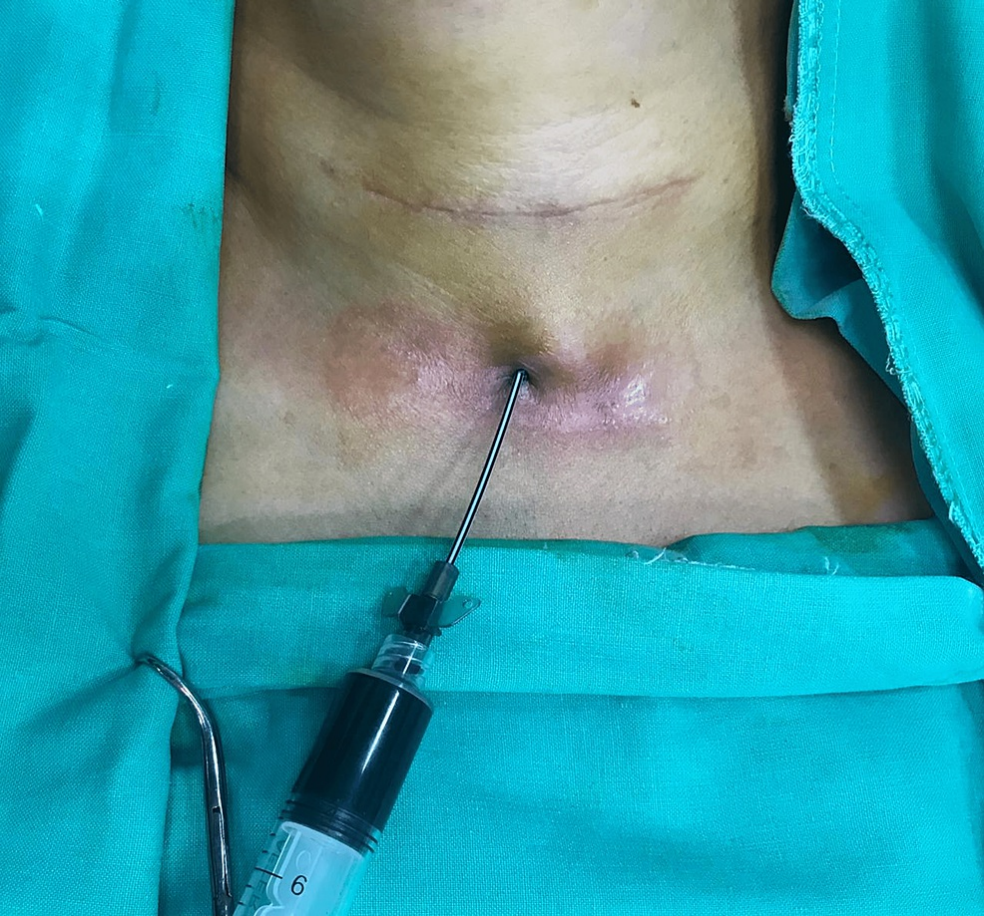
Injection of methylene blue at the site of the fistula

**Figure 5:**
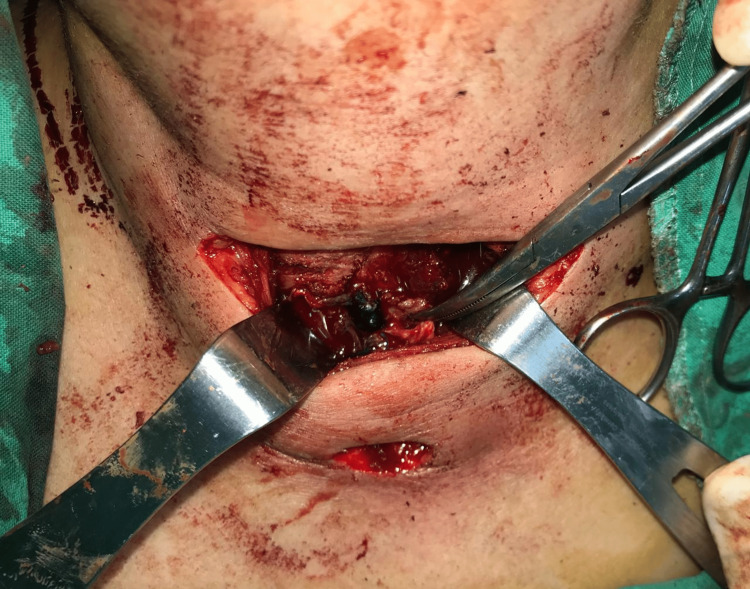
The colored oxidized regenerated cellulose gauze at the right paratracheal space (thyroid bed)

**Figure 6:**
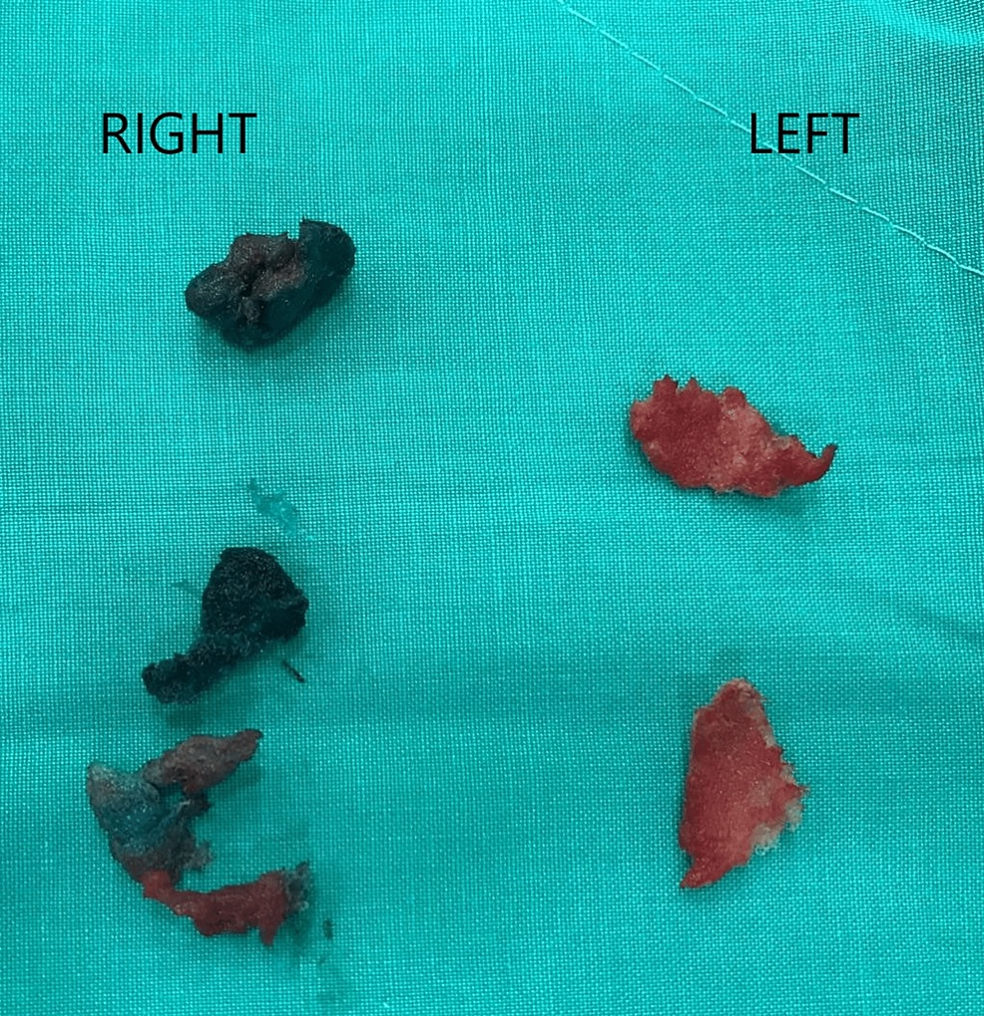
Oxidized regenerated cellulose gauze colored blue by the methylene blue

**Figure 7:**
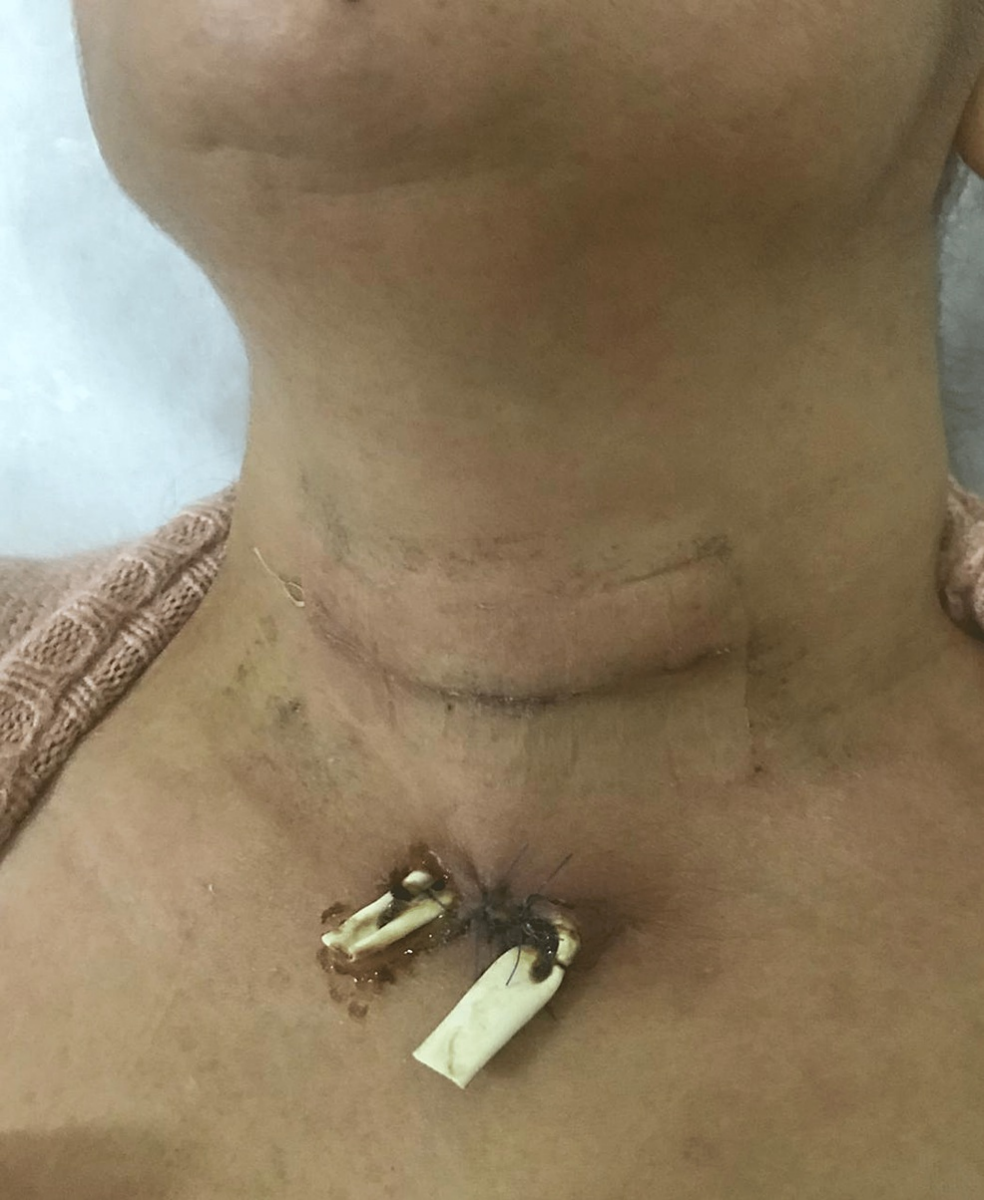
Wound closed with the placement of Penrose drain

Further review of the operative report revealed that ORC had been used for hemostasis on both sides at the conclusion of the thyroidectomy surgery. Given the possibility of retained hemostatic material causing chronic inflammation and infection, the decision was made to explore the neck for a persistent non-healed fistula. The patient was taken to the operating room, where the fistula opening was probed, and methylene blue was injected to identify the tract and the feeding collection (Figure 4). The previous neck incision was reopened, and dissection was carried out carefully to drain the deep collection behind the left sternocleidomastoid muscle, and culture was obtained from the purulent fluid. The left paratracheal space was accessed, revealing nonresorbed pieces of ORC. The fistula tract was then traced from the midline to the right paratracheal space (Figure 5), thyroid bed where retained ORC gauze colored blue by the methylene blue (Figure 6) reflecting the origin and cause of the persistent fistula. All hemostatic materials were removed, and several cultures were obtained. The whole sinus tract was excised, and the outside opening was debrided. The wound was closed primarily, and a Penrose drain was placed (Figure 7). Cultures revealed negative results for regular, aerobic, anaerobic, fungal, and tuberculosis microorganisms.

## Discussion

Chronic sinus tract formation in the neck following thyroid surgery has been reported in the literature [7,8]. Several risk factors have been identified, including infection, use of nonabsorbable sutures, and the presence of a foreign body. Diabetes mellitus has also been linked to an increased risk of infection and sinus formation [9]. In recent years, the use of ORC as a hemostatic agent has become increasingly common in surgery, including thyroid surgery. While ORC is supposed to dissolve completely within one to two weeks [10], there have been reports of retained material causing complications such as infection and sinus formation. The present case report is unique in that it highlights the potential risk of chronic sinus tract formation related to the use of nonresorbed ORC mesh in thyroid surgery.

Maintaining a sterile environment during surgery is critical to minimizing the risk of infection and other complications. To achieve this, healthcare providers assess the sterility of equipment used in the initial surgery (including the drain and ORC), remain vigilant to detect and address breaks in sterility during the procedure, and administer preoperative antibiotics. These important steps help optimize patient outcomes by reducing the likelihood of postoperative complications. This finding is consistent with previous reports of retained material causing complications following surgery and underscores the need for healthcare providers to monitor patients carefully for signs of infection and other complications [11,12]. While ORC is generally considered safe and effective, it is important to recognize the potential risks associated with its use. Patients should be informed about the potential risks and benefits of ORC use, and healthcare providers should take appropriate measures to minimize the risk of complications. The present case report adds to the growing body of evidence highlighting the potential risk of postoperative complications related to the use of ORC mesh. By working together, healthcare providers and patients can help minimize the risk of complications and ensure the best possible outcomes following surgery. Further studies are needed to better understand the risk factors associated with chronic sinus tract formation and other complications following thyroid surgery with the use of ORC and to identify strategies for minimizing these risks.

## Conclusions

This is the first report of the occurrence of chronic sinus formation secondary to the use of ORC during thyroid surgery. This highlights the need for both surgeons and patients to be aware of this possible complication. While infection and nonabsorbable sutures have commonly been implicated as causative factors, our presented case suggests that nonresorbable ORC mesh used as a hemostatic agent during surgery may contribute to chronic sinus formation. Effective management of this condition requires a comprehensive neck exploration, meticulous removal of all retained material, and excision of the sinus tract. These findings significantly advance our understanding of the potential mechanisms underlying neck sinus formation post-thyroidectomy and highlight the importance of vigilant monitoring and timely intervention in managing this infrequent yet significant complication. Further research is needed to explore the risk factors and preventive measures for neck sinus formation, ultimately enhancing the safety and outcomes of thyroidectomy procedures.
